# Skill-Based Virtual Reality Therapy in High-Impact Chronic Pain: 2-Year Follow-Up Results From a Secondary Analysis of a Randomized Controlled Trial

**DOI:** 10.2196/90688

**Published:** 2026-04-23

**Authors:** Todd Maddox, Josh Sackman, Robert Bonakdar, Roselani Maddox, Beth D Darnall

**Affiliations:** 1 AppliedVR Van Nuys, CA United States; 2 Scripps Health La Jolla, CA United States; 3 Stanford Medicine Palo Alto, CA United States

**Keywords:** high-impact chronic pain, HICP, low-impact chronic pain, LICP, virtual reality, VR, digital health

## Abstract

**Background:**

High-impact chronic pain (HICP) involves substantial interference in functioning, affects 8.5% of the population, and leads to higher health care costs relative to low-impact chronic pain (LICP). Behavioral interventions such as virtual reality (VR) offer scalable and accessible treatment, but testing is needed to ensure durable effectiveness in HICP. We conducted a secondary analysis of the largest real-world dataset for a therapeutic skill-based VR vs a sham VR control to test treatment efficacy in HICP vs LICP. Relative to LICP, we found significantly larger (and clinically meaningful; ie, ≥2 points) pain interference and pain intensity reductions for HICP at end of treatment and 1 year posttreatment. End-of-treatment reduction in pain interference reclassified 70% (114/163) of participants with HICP as LICP, and this improvement held at 1 year posttreatment (104/155, 67%).

**Objective:**

This study examined the effectiveness of a 56-session skill-based VR therapy in HICP at 2 years posttreatment and compared the effects with those on LICP.

**Methods:**

We conducted a secondary analysis of the skill-based VR sample (536/1067, 50.2%) at 2 years posttreatment from a randomized controlled trial involving an in-home chronic low back pain sample that was recruited and tested online and was diverse (female: 411/536, 77%; non-White individuals: 166/536, 31%; high school or lower educational level: 102/536, 19%; mean age 50.8 years) and had clinically severe pain at baseline (intensity=6.6; interference=6.2; 42% with severe or complete disability). Focusing on the skill-based VR participants (536/1067, 50.2%) and using a validated approach, we classified participants at baseline as either HICP (baseline Brief Pain Inventory pain interference score>7) or LICP (baseline Brief Pain Inventory pain interference score<7). Clinical effectiveness was examined using a general linear model at 2 years posttreatment relative to baseline with the primary outcomes of pain interference and pain intensity.

**Results:**

Participants with HICP (192/536, 35.8%) reported superior reductions in pain interference, pain intensity, sleep disturbance, and physical disability (*P*<.001 in all cases) at 2 years posttreatment compared to participants with LICP (344/536, 64.2%). Participants with HICP had clinically meaningful (≥2-point) reductions in pain interference (mean 3.1, 95% CI 2.66-3.54; effect size=1.12) and pain intensity (mean 2.6, 95% CI 2.18-3.02; effect size=1.01) at 2 years posttreatment. Importantly, reduced pain interference scores at 2 years posttreatment reclassified 71.1% (106/149) of the participants with HICP as LICP. No serious adverse events or side effects were reported.

**Conclusions:**

Patients with HICP experience severe pain that drives high health care use. The skill-based VR therapy demonstrated durable reductions in pain and related outcomes 2 years posttreatment, with the largest benefits observed in the HICP subgroup. These results suggest that a skill-based, VR-delivered therapy produces durable effects in patients with HICP, a population that is frequently overmedicalized and undertreated with behavioral interventions. These findings suggest that home-based VR-delivered therapy offers a scalable treatment option for this underserved population.

## Introduction

### Background and Rationale

High-impact chronic pain (HICP) is defined as pain lasting at least 3 months that also causes a major limitation in daily functioning—such as the inability to attend school, work outside the home, or complete household tasks [1-4]. In 2023, nearly 25% of adults reported experiencing chronic pain within the previous 3 months, and 8.5% reported high levels of chronic pain that frequently restricted their ability to engage in work or life activities (classified as HICP) [3,5]. Importantly, the prevalence of HICP grew from 7.4% in 2019 to 8.5% in 2023 and continues to grow on an annual basis [5]. In fact, a recent 2025 publication [6] suggested that factors such as long COVID, sedentary lifestyles, and reduced health care access during and after the COVID-19 pandemic have contributed to the record high and growing levels of HICP. Critically, HICP, relative to low-impact chronic pain (LICP), has increased health care use and costs. Patients with HICP are more likely to receive opioid prescriptions at 5 times the morphine-equivalent daily dose and are 3 times more likely to undergo surgery and have emergency department visits [3,7-10]. Recognizing these burdens, the US Department of Health and Human Services highlighted the need to prioritize HICP in the 2016 National Pain Strategy [11]. Given the increased prevalence and increased pain treatment and health care use patterns, patients with HICP are ideal candidates for low-risk and accessible in-home interventions such as virtual reality (VR)–delivered therapy, but it is important to verify strong treatment effectiveness and patient engagement with these immersive therapeutics in HICP. In-person therapies such as cognitive behavioral therapy are moderately effective for HICP [12,13], but to date, only 1 published study has examined treatment effectiveness and patient engagement with an immersive therapeutic [14].

In a recent large-sample randomized controlled trial in chronic lower back pain [15,16], we found durable efficacy and statistical superiority of a Food and Drug Administration–authorized in-home 56-session skill-based VR therapy relative to a 56-session active sham VR control at end of treatment and 1 year posttreatment. With durable efficacy and superiority to the sham control established, we conducted a secondary analysis [14] of the skill-based VR group and found significantly larger (and clinically meaningful; ie, ≥2-point reductions [17,18]) in pain interference and pain intensity (primary end points) for participants with HICP than for participants with LICP at end of treatment and 1 year posttreatment. Importantly, end-of-treatment reduction in pain interference among patients with HICP reclassified 70% of them as LICP, and this improvement held at 1 year posttreatment (67%). Significantly larger reductions in sleep disturbance and physical disability (secondary end points) were found for HICP compared to LICP. No differences were found for HICP vs LICP in device engagement or device usability, both of which were high.

### Objectives

The aim of this report was to conduct the first-ever examination of long-term effectiveness of the skill-based VR-delivered therapy in patients with HICP at 2 years posttreatment. As in previous work [14], we used the validated classification approach for the Brief Pain Inventory (BPI) pain interference score [19,20] of 7 or more to classify participants as either HICP or LICP at baseline and at 2 years posttreatment to determine individual change in classification category [21]. Specifically, within the skill-based VR group, we compared treatment response for HICP vs LICP at 2 years posttreatment relative to baseline. We hypothesized that the skill-based VR-delivered therapy would lead to strong and durable pain reductions (primary end points), as well as durable reductions in sleep disturbance and physical disability (secondary end points) in patients with HICP.

## Methods

### Patient and Public Involvement

There was no patient or public involvement in the design, conduct, or reporting of this secondary analysis. The primary trial from which these data were derived was designed and conducted by the study investigators.

### Trial Design, Trial Setting, and Participant Eligibility

The methods, CONSORT (Consolidated Standards of Reporting Trials) diagram, and analyses at end of treatment and 1 year posttreatment for the primary decentralized randomized controlled trial are published elsewhere [15,16]. This secondary analysis is reported in accordance with the CONSORT 2025 statement [22] and CONSORT-EHEALTH (Consolidated Standards of Reporting Trials of Electronic and Mobile Health Applications and Online Telehealth) guidelines [23], and there were no changes to the methods after trial commencement. Although the focus of this report is on the skill-based VR arm of the study, in particular a 2-year durability test of the skill-based VR treatment advantage in the HICP group, for completeness, we briefly describe some of the trial details, including the sham VR group. A double-blind, randomized placebo-controlled trial was conducted in a national community sample of 1067 individuals with self-reported chronic lower back pain for at least 3 months with an average BPI [19,20] pain intensity and pain interference score of 4 or higher for the previous month on a scale from 0 to 10 points. The sample had clinically severe pain (baseline pain intensity=6.6; baseline pain interference=6.2; disability=42% severe or complete) and was demographically diverse (female: 411/536, 77%; non-White individuals: 166/536, 31%; high school or lower educational level: 102/536, 19%; mean age 50.8 years).

### Intervention and Randomization

Participants were randomized 1:1 to either (1) a 56-session immersive 3D pain relief skill-based VR therapy or (2) a sham VR control (2D nature videos delivered in a VR headset with no pain relief skill content) and received the device (VR device that includes either the skills-based VR therapy or the sham VR therapy) via mail. The skill-based VR therapy included a fixed sequence of 56 daily VR sessions that delivered pain neuroscience education along with evidence-based self-regulatory strategies commonly taught in behavioral pain treatments, such as cognitive behavioral therapy and mindfulness training. The sham VR condition was designed to replicate the look and feel of the skill-based VR therapy while omitting 3D immersion, spoken content, and pain relief skills. Instead, it consisted of a fixed sequence of 56 nature videos in 2D accompanied by neutral music.

### Relevant Self-Report Outcome Measures

These are all widely used and well-validated measures that are effective for measuring changes in chronic lower back pain and relevant comorbidities following VR-delivered therapy.

#### Demographics

Age, gender, race, ethnicity, annual household income, height, weight, and pain comorbidities were collected.

#### BPI: Pain Interference

The BPI pain interference scale measures how much pain interfered with 7 domains of daily life (enjoyment of life, general activity, mood, normal work, relationships with other people, sleep, and walking ability) over the previous 24 hours using a numeric rating scale from 0 to 10 [20]. The scores on the 7 items are averaged to generate a global pain interference score. Five administrations of the BPI pain interference scale were included (1 per day) during the 5 days following initial baseline administration and prior to the start of therapy, with a requirement that at least 2 be completed. These were averaged with the baseline BPI score to obtain the overall baseline BPI pain interference score. The BPI pain interference scale was also administered at end of treatment and 1 and 2 years posttreatment.

#### BPI: Pain Intensity

The BPI pain intensity scale uses a single item to measure pain intensity over the previous 24 hours using a numeric rating scale from 0 to 10 [20]. Five administrations of the BPI pain intensity scale were included (1 per day) during the 5 days following initial baseline administration and prior to the start of therapy, with a requirement that at least 2 be completed. These were averaged with the baseline BPI score to obtain the overall baseline BPI pain intensity score. The BPI pain intensity scale was also administered at end of treatment and 1 and 2 years posttreatment.

#### Patient-Reported Outcomes Measurement Information System Sleep Disturbance (Version 8b)

The Patient-Reported Outcomes Measurement Information System (PROMIS) Sleep Disturbance tool is an 8-item survey that assesses an individual’s perception of sleep quality, sleep depth, and restoration associated with sleep over the previous 7 days [24]. Raw scores on the PROMIS Sleep Disturbance survey are converted to T scores, which range from 28.9 to 76.5. This measure was administered at baseline, end of treatment, and 1 and 2 years posttreatment.

#### PROMIS Depression (Version 8b)

The PROMIS Depression tool is an 8-item survey that assesses the frequency and severity of depressive symptoms over the previous 7 days [25]. It covers topics such as feelings of worthlessness, hopelessness, and loss of interest in activities. Raw scores on the PROMIS Depression survey are converted to T scores, which range from 37.1 to 81.1. This measure was administered at baseline, end of treatment, and 1 and 2 years posttreatment.

#### Oswestry Disability Index (Version 2.1b)

The Oswestry Disability Index (ODI) is a 10-item survey that assesses how low back pain affects one’s ability to manage in everyday life [26]. Scores range from 0 to 100 and reflect the level of disability. This measure was administered at baseline, end of treatment, and 1 and 2 years posttreatment.

### Harms

Harms were assessed and reported for the primary randomized controlled trial [15]. No serious adverse events related to the VR intervention were reported. Harms were not separately reassessed in this secondary analysis, which focused on long-term efficacy outcomes by pain impact subgroup.

### Ethical Considerations

With respect to human subject research ethics review, exemptions, and approvals, the study protocol (approval 132166) was approved by the WCG Institutional Review Board (Puyallup, Washington) in December 2021 and followed the CONSORT reporting guidelines. The randomized controlled trial was prospectively registered with ClinicalTrials.gov on January 31, 2022 (ClinicalTrials.gov identifier: NCT05263037). The first participant was enrolled on February 14, 2022. All participants provided informed consent, and study data were collected from February 14, 2022, to October 31, 2024 (2 years posttreatment). Participant identification was protected with a unique study identification number. Data were received electronically through Curebase, secured in the database, and stored with double password protection. All study data were anonymized and deidentified. Study participants received US $210 for the completion of all study surveys up to day 56 (end of treatment), an additional US $90 for completing the 1-year posttreatment surveys, and an additional US $90 for completing the 2-year posttreatment follow-up surveys. No images in the manuscript or supplementary materials contain identifiable individual participants.

### Statistical Analysis Methods

The standardized mean differences between the skill-based VR HICP and LICP groups were calculated. Between-group comparisons (HICP vs LICP) were conducted using a general linear model with age, sex, and BMI as covariates. First, we evaluated the skill-based VR therapy effects in HICP vs LICP on the 2 primary end points (BPI pain interference and BPI pain intensity reduction from baseline to 2 years posttreatment). The Bonferroni-adjusted statistical significance level for comparing groups was set at a 2-sided value of .03 to adjust for 2 comparisons. Next, we evaluated the skill-based VR therapy effects in HICP vs LICP on the 3 secondary end points (PROMIS Sleep Disturbance, PROMIS Depression, and ODI reduction from baseline to 2 years posttreatment). The Bonferroni-adjusted statistical significance level for comparing groups was set at a 2-sided value of .02 to adjust for 3 comparisons.

## Results

### Participant Characteristics

**Figure 1:**
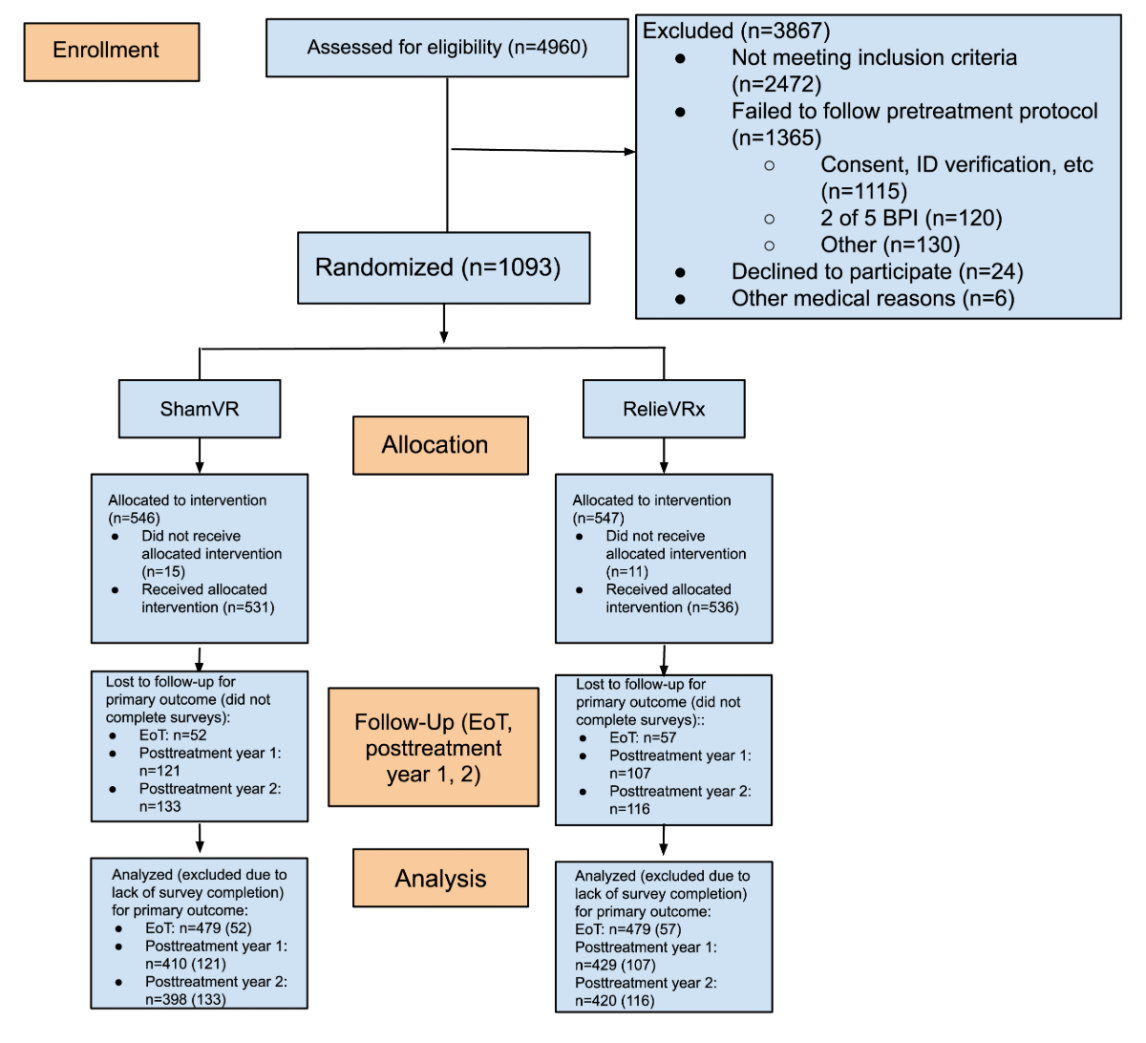
CONSORT (Consolidated Standards of Reporting Trials) participant flow diagram for the full randomized controlled trial comparing skill-based virtual reality (VR) with sham VR control in patients with chronic low back pain (participant flow for end of treatment and 1 and 2 years posttreatment included).

Complete demographics and baseline clinical measures, as well as full results for the skill-based VR group analysis of the primary and secondary end points at end of treatment and 1 year posttreatment by pain impact group (LICP vs HICP) are published and accessible via open source [15,16]. The CONSORT participant flow diagram for the skill-based VR and sham VR groups is presented in Figure 1. Table 1 shows the baseline demographic and clinical characteristics by pain impact group (LICP vs HICP) for the skill-based VR therapy participants. A total of 35.8% (192/536) were classified as HICP. The HICP group had greater racial diversity (*P*=.005); lower annual household income (*P*<.001); higher BMI (*P*=.01); and significantly greater levels of symptom severity across all variables, with *P* value group differences ranging from .01 to <.001. Two-year posttreatment survey completion was high, with 88.1% (420/477) of skill-based VR therapy participants who completed end-of-treatment surveys completing the 2-year posttreatment survey. No differences in demographic or baseline clinical variables were observed between dropouts and responders within the skill-based VR HICP or LICP subgroups. In addition, the pattern of demographic and baseline clinical variable differences observed between the skill-based VR HICP and LICP subgroups did not differ for dropouts vs responders.

**Table 1:** Participant demographic characteristics and select baseline measures in the modified intent to treat analysis for the subgroup of participants who completed the skill-based virtual reality (VR) therapy in a randomized controlled trial that compared skill-based VR with a sham VR control in patients with chronic low back pain.

			Skill-based VR—LICP^a^ (n=344)		Skill-based VR—HICP^b^ (n=192)		*P* value^c^
**Gender, n (%)**							.03
	Female	253 (73.5)		158 (82.3)			
	Male	90 (26.2)		34 (17.7)			
	Nonbinary	1 (0.3)		0 (0)			
**Race and ethnicity, n (%)**							.005
	American Indian or Alaska Native	3 (0.9)		1 (0.5)			
	Asian or Pacific Islander	7 (2)		5 (2.6)			
	Black or African American	34 (9.9)		47 (24.5)			
	Hispanic or Latino	11 (3.2)		7 (3.6)			
	Multiracial	29 (8.4)		22 (11.5)			
	White	260 (75.6)		110 (57.3)			
Age (y), mean (SD)			50.9 (14.2)		49.5 (11.9)		.23
**Annual household income (US $), n (%)**							<.001
	<60,000	181 (52.6)		133 (69.3)			
	≥60,000	163 (47.4)		59 (30.7)			
**Number of pain comorbidities, n (%)**							.52
	0	140 (40.7)		75 (39.1)			
	≥1	182 (52.9)		110 (57.3)			
	Nonreported	22 (6.4)		7 (3.6)			
BMI (kg/m^2^), mean (SD)			30.4 (7.7)		33.9 (17.8)		.01
BPI^d^ pain intensity (0-10), mean (SD)			5.9 (1.2)		7.9 (1.0)		<.001
BPI pain interference (0-10), mean (SD)			5.2 (1.4)		8.1 (0.8)		<.001
PROMIS^e^ Anxiety, mean (SD)			55.1 (8.8)		57.9 (9.5)		<.001
PROMIS Sleep Disturbance, mean (SD)			58.7 (6.7)		64.4 (6.5)		<.001
PROMIS Depression, mean (SD)			54.0 (8.6)		56.7 (10.2)		.002
ODI^f^, mean (SD)			36.0 (13.2)		51.3 (14.7)		<.001

^a^LICP: low-impact chronic pain.

^b^HICP: high-impact chronic pain.

^c^*P* value from 2-tailed *t* test or Fisher exact test comparing LICP to HICP.

^d^BPI: Brief Pain Inventory.

^e^PROMIS: Patient-Reported Outcomes Measurement Information System.

^f^ODI: Oswestry Disability Index.

### Skill-Based VR Therapy Pain Impact Group Differences at 2 Years Posttreatment

#### Primary End Points

**Figure 2:**
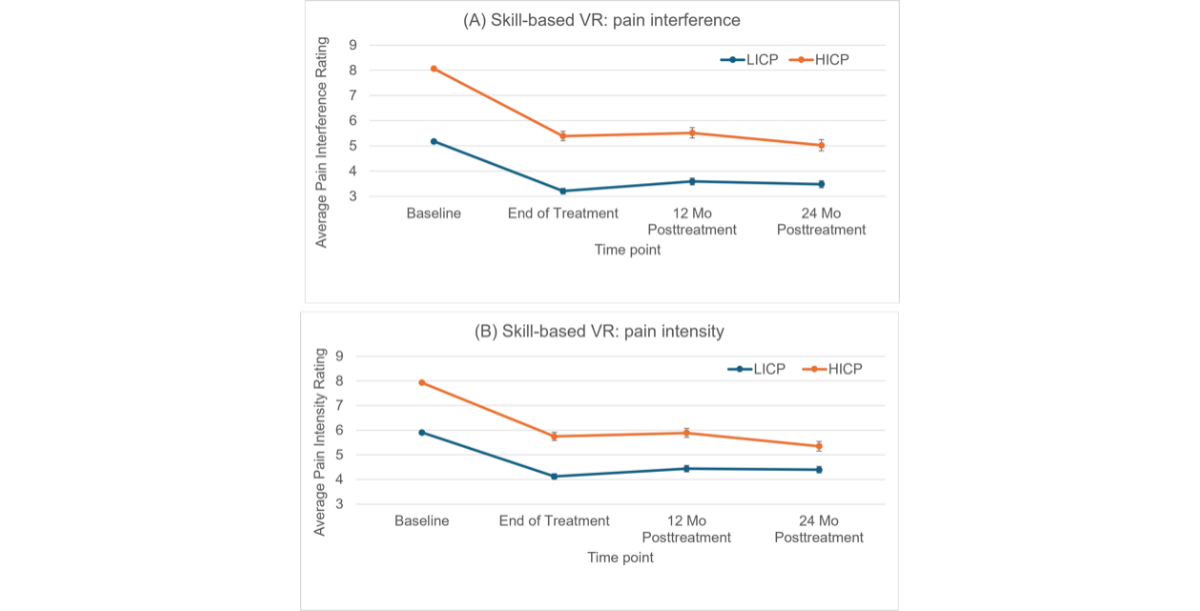
Average (A) pain interference and (B) pain intensity reductions (with error bars) from baseline to end of treatment and 1 and 2 years posttreatment for the subgroup of participants who completed the skill-based virtual reality (VR) therapy (subdivided into high-impact chronic pain [HICP] and low-impact chronic pain [LICP] groups) from a randomized controlled trial comparing skill-based VR with a sham VR control in patients with chronic low back pain.

Table 2 shows the mean reductions, 95% CIs, and effect sizes for BPI pain interference and BPI pain intensity from baseline to 2 years posttreatment in the skill-based VR therapy group separately for the HICP and LICP groups. The average pain interference reduction was statistically larger in the HICP group (mean 3.1, 95% CI 2.66-3.54; within-group effect size=1.12) than in the LICP group (mean 1.7, 95% CI 1.45-1.95; within-group effect size=0.79; *P*<.001; between-group effect size=0.60). Interestingly, at 2 years posttreatment, the average pain interference score of participants with HICP was classified in the LICP range (2-year posttreatment average=5.02), with 71.1% (106/149) of individual participants with HICP at 2 years posttreatment reclassified as LICP. The average pain intensity reduction was statistically larger in the HICP group (mean 2.6, 95% CI 2.18-3.02; within-group effect size=1.01) than in the LICP group (mean 1.5, 95% CI 1.24-1.76; within-group effect size=0.68; *P*<.001; between-group effect size=0.48). Although they have been published elsewhere [14], Table 2 also includes the primary end point results from 1 year posttreatment for comparison purposes. The 1-year posttreatment findings mirror those from 2 years posttreatment, suggesting that the HICP advantage is robust and durable. Figure 2 shows the average pain interference (panel A) and average pain intensity (panel B) ratings for the HICP and LICP groups at baseline, end of treatment, and 1 and 2 years posttreatment, providing a visual of the trajectory of pain ratings over time.

**Table 2:** Primary and secondary end point reductions from baseline to 2 years posttreatment and from baseline to 1 year posttreatment for the subgroup of participants who completed the skill-based VR therapy in a randomized controlled trial comparing skill-based VR with a sham VR control in patients with chronic low back pain.

		HICP^a,b^	LICP^b,c^	Between-group effect size	*P* value: HICP vs LICP
**2 years posttreatment relative to baseline**					
	BPI^d^ Pain Interference	3.1; 2.66 to 3.54; (1.12)	1.7; 1.45 to 1.95; (0.79	0.60	<.001
	BPI Pain Intensity	2.6; 2.18 to 3.02; (1.01)	1.5; 1.24 to 1.76; (0.68)	0.48	<.001
	PROMIS^e^ Sleep Disturbance	7.8; 6.25 to 9.35; (0.80)	4.1; 3.17 to 5.03; (0.53)	0.43	<.001
	PROMIS Depression	0.9; –0.9 to 2.70; (0.08)	1.9; 0.93 to 2.87; (0.24)	0.11	.34
	Oswestry Disability Index	14.7; 11.87 to 17.53; (0.84)	8.3; 6.59 to 10.01; (0.58)	0.42	<.001
**1 year posttreatment relative to baseline**					
	BPI Pain Interference	2.5; 2.2 to 2.8; (1.04)	1.6; 1.35 to 1.85; (0.73)	0.45	<.001
	BPI Pain Intensity	2.0; 1.65 to 2.35; (0.93)	1.5; 1.26 to 1.74; (0.71)	0.30	.003
	PROMIS Sleep Disturbance	6.9; 5.46 to 8.34; (0.74)	4.5; 1.63 to 5.37; (0.56)	0.33	.001
	PROMIS Depression	1.5; –0.43 to 3.43; (0.09)	1.9; 1.01 to 2.79; (0.25)	0.08	.43
	Oswestry Disability Index	12.7; 9.93 to 15.47; (0.69)	8.8; 7.13 to 10.47; (0.60)	0.23	.02

^a^HICP: high-impact chronic pain.

^b^Mean; 95% CIs (within-group effect size) point reduction from baseline to 2-years posttreatment or 1-year posttreatment.

^c^LICP: low-impact chronic pain.

^d^BPI: Brief Pain Inventory

^e^PROMIS: Patient-Reported Outcomes Measurement Information System.

#### Secondary End Points

Table 2 also shows the mean reductions, 95% CIs, and effect sizes for the PROMIS Sleep Disturbance, PROMIS Depression, and ODI from baseline to 2 years posttreatment in the skill-based VR therapy group separately for the HICP and LICP groups. The average sleep disturbance reduction was statistically larger in the HICP group (mean 7.8, 95% CI 6.25-9.35; within-group effect size=0.80) than in the LICP group (mean 4.1, 95% CI 3.17-5.03; within-group effect size=0.53; *P*<.001; between-group effect size=0.43). The average depression reduction was 0.9 (95% CI −0.90 to 2.70; within-group effect size=0.08) in the HICP group and 1.9 (95% CI 0.93-2.87; within-group effect size=0.24) in the LICP group and did not differ statistically (*P*=.34; between-group effect size=0.11). The average disability reduction was statistically larger in the HICP group (mean 14.7, 95% CI 11.87-17.53; within-group effect size=0.84) than in the LICP group (mean 8.3, 95% CI 6.59-10.01; within-group effect size=0.58; *P*<.001; between-group effect size=0.42). Although they have been published elsewhere [14], Table 2 also includes the secondary end point results from 1 year posttreatment for comparison purposes. The 1-year posttreatment findings mirror those from 2 years posttreatment, suggesting that the HICP advantage is robust and durable.

## Discussion

### Principal Findings

In this first-ever examination of treatment durability to 2 years posttreatment for a 56-session skill-based VR-delivered therapy for chronic low back pain, we found that the HICP group had clinically meaningful pain interference and pain intensity reductions that were statistically superior to those in the LICP group, with large within-group effect sizes. Most participants with HICP (106/149, 71%) were reclassified as LICP at 2 years posttreatment. We also found larger sleep disturbance and physical disability reductions for HICP over LICP at 2 years posttreatment. These findings are consistent with the hypothesis that patients with HICP would demonstrate strong and durable treatment response, confirming and extending our prior findings at 1 year posttreatment [14].

Patients with HICP have higher health service use (including more frequent primary care visits, specialist consultations, and emergency department use and greater reliance on diagnostic imaging and interventional procedures) [7-10], greater risks from various pain treatments—including medication side effects, complications from invasive procedures, and potential misuse of medications—and only modest clinical improvement relative to patients with LICP [27-29]. These elevated risks are particularly concerning given the diminishing marginal returns of repeated or escalated medical interventions in this population, suggesting a mismatch between treatment intensity and therapeutic benefit [1]. HICP is also more common among women, older adults, people with lower socioeconomic status, and rural residents [3,30], likely reflecting a complex interaction between biological vulnerability, psychosocial stressors, reduced access to comprehensive pain care, and structural inequities in health care delivery. Although these groups have long been known to experience more severe chronic pain, the HICP classification offers a validated way to examine pain impacts and assess treatment effectiveness [21]. Importantly, this classification moves beyond simple pain intensity metrics by incorporating functional impact, thereby providing a more clinically meaningful framework for stratifying patients and tailoring interventions [21]. Data show that patients with HICP are often overmedicalized and undertreated with behavioral approaches [31]. This imbalance may perpetuate a cycle in which patients become increasingly dependent on biomedical interventions while missing opportunities to develop adaptive self-regulation and coping skills. There is evidence suggesting that an HICP treatment model should prioritize behavioral medicine interventions at the outset of pain treatment to minimize patient risks and enhance both pain and health outcomes [32], possibly preventing chronicity by targeting maladaptive learning processes such as fear avoidance and pain catastrophizing before they become entrenched. In-person behavioral medicine approaches such as cognitive behavioral therapy have demonstrated modest efficacy for HICP [12,13], but access is often poor because of the multisession and therapist-led nature of cognitive behavioral therapy [33]. Additional barriers include geographic limitations, long wait times, cost, and stigma associated with seeking psychological treatment for pain [32]. In addition, effectiveness can fluctuate based on the quality of the therapist [34], thus introducing inconsistency in treatment delivery, which may attenuate outcomes and complicate efforts to scale evidence-based care. The skill-based VR intervention evaluated in this study offers high-quality, consistent therapy from session to session that is available on demand in the home, thus helping address the access and therapist quality issues [15]. By standardizing therapeutic content while maintaining interactivity and engagement, VR-based delivery may reduce variability and enhance treatment fidelity across diverse patient populations [32]. The skill-based VR therapy is also easy to use, as evidenced by its A+ rating on the System Usability Scale [14]. Importantly, this A+ rating was observed across several sociodemographic factors (age, gender, race and ethnicity, and socioeconomic status) [35] and for both HICP and LICP [14], suggesting that the intervention is broadly accessible and does not exacerbate existing disparities in digital health engagement. The results of this study combined with those of our previous study [14] suggest that the patients with chronic lower back pain who are most impacted and symptomatic (ie, patients with HICP) reaped the largest short-term and long-term benefits from the skill-based VR therapy. This pattern is consistent with a “greater room for improvement” effect but may also reflect heightened responsiveness to interventions that directly target central pain modulation and cognitive-emotional processes [36]. The finding that 71.1% (106/149) of patients with HICP were reclassified as LICP at 2 years posttreatment is also noteworthy. Future research may examine whether most patients with HICP who were reclassified as LICP also exhibit expected reductions in health care use and associated medical treatment risks. Longitudinal analyses linking classification changes to downstream health care costs, opioid use, and procedural interventions would provide critical evidence for the broader health-economic value of this approach.

The existing literature suggests that user experience features such as interactivity, gamification, and virtual embodiment systematically modulate how much pain relief people obtain from VR, with more engaging and embodied designs generally producing larger analgesic effects [37-39]. Although the skill-based VR therapy contained some elements of interactivity, gamification, and embodiment, it was primarily designed as a multimodal program drawing on cognitive behavioral strategies, meditation, mindfulness, biofeedback, education, and interoceptive awareness and control [15]. Collectively, these results suggest that complex patients with HICP gain significant advantages from this self-delivered multimodal therapy. Even so, building on the existing literature, future iterations of the therapy should explore incorporating more interactivity, gamification, and virtual embodiment as this could enhance the effectiveness of the therapy beyond the current level.

### Limitations and Strengths

Several limitations should be considered when interpreting these findings. First, although the HICP vs LICP classification is empirically derived, participants could not be randomized, and the results remain correlational. Second, unmeasured factors may be causal, including pain-related and non–pain-related comorbidities. Greater characterization of the diagnoses and conditions underlying the HICP group (eg, trauma history, disability status, and cancer or palliative care) and of concurrent treatments over the 2-year period (eg, opioid use, injections, and surgery) would strengthen interpretation and should be addressed in future research. Third, chronic lower back pain was self-reported and not clinically confirmed. Finally, because the study focused on chronic lower back pain, the findings may not generalize to other populations. Despite these limitations, we also note several study strengths that contextualize its value. First, these results come from the largest real-world community sample dataset for therapeutic skill-based VR (n=536) with a large HICP subset (n=192). Second, 2-year participant retention was high, with 88.1% (420/477) of skill-based VR therapy participants who completed end-of-treatment surveys completing the 2-year posttreatment survey. Finally, we tested heterogeneity of treatment effect across a factor that is broadly applicable and relevant to clinicians (ie, HICP vs LICP).

### Conclusions

This secondary analysis provides the first evidence of 2-year durability of a skill-based VR-delivered therapy in HICP, demonstrating clinically meaningful and sustained benefits in a population that is often overmedicalized and undertreated with behavioral approaches. The finding that most participants with HICP were reclassified as LICP at 2 years posttreatment has potential implications for reducing health care use and improving quality of life in this high-need population. These results support continued investigation of home-based, self-administered VR-delivered behavioral therapy as a scalable, accessible, and effective treatment option for patients with HICP. Future research should examine whether HICP reclassification is associated with reductions in health care use and should evaluate the therapy across other chronic pain conditions.

## Data Availability

The datasets generated or analyzed during this study are available from the corresponding author on reasonable request.

## Appendix

Multimedia Appendix 1 CONSORT checklist.

## Abbreviations

BPI: Brief Pain Inventory
CONSORT: Consolidated Standards of Reporting Trials
CONSORT-EHEALTH: Consolidated Standards of Reporting Trials of Electronic and Mobile Health Applications and Online Telehealth
HICP: high-impact chronic pain
LICP: low-impact chronic pain
ODI: Oswestry Disability Index
PROMIS: Patient-Reported Outcomes Measurement Information System
VR: virtual reality
